# Transient Global Amnesia After Ablation of the Left Lateral Accessory Pathway

**DOI:** 10.1016/s0972-6292(16)30482-x

**Published:** 2012-04-30

**Authors:** Chen-Ying Chou, Ching-Pei Chen, Ching-Hui Huang

**Affiliations:** Department of Cardiology, Changhua Christian Hospital

**Keywords:** transient global amnesia, ablation, paroxysmal supraventricular tachycardia

## Abstract

Transient global amnesia (TGA) could be encountered in many situations even during invasive procedures. In ablation therapy for arrhythmia, there was only one reported case in the ablation of premature ventricular beats. We report a 31-year-old man having paroxysmal supraventricular tachycardia who underwent TGA at the end of ablation and recovered quickly after 8-9 hours later. Long-term follow-up showed no neurologic deficits for 8 months.

## Introduction

Transient global amnesia (TGA) often has an abrupt onset. It may occur during strenuous physical activity, emotional stress, or prolonged abnormal head posture, as well as during medical procedures such as cerebral and coronary angiography or rarely, as recently reported in the literature, during the ablation for premature ventricular beats arising from the right coronary cusp [1]. Here, we describe a male patient who experienced TGA after ablation therapy for paroxysmal supraventricular tachycardia.

## Case Report

The patient was a 31-year-old man who had no previous medical history of interest. Repeated palpitations were noted in the past 3 months. Paroxysmal supraventricular tachycardia (Figure 1A) was diagnosed, and he was scheduled to undergo ablation.

During the procedure, electrophysiologic catheters were placed into the coronary sinus, high right atrium, His-bundle area, and the right ventricular apex through jugular and femoral veins. Supraventricular tachycardia was observed using a left lateral accessory pathway for retrograde conduction. A heparin bolus was administered, and activated clotting time was determined to be 256 seconds. Thereafter, we performed ablation in the lateral wall of left ventricle. Total procedural time was 40 minutes (Figure 1B).

At the end of the procedure, the patient felt strange in his surroundings. He could not remember what happened after hospitalization. So, he was confused as to why he was in the catheterization laboratory and why he had undergone the procedure. Although we answered and explained to him, he forgot very soon and asked the same questions again and again. He was able to recognize the persons he knows and able to tell that he is in the hospital. Although there was no communication problem, he had difficulty remembering things that we just talked. His vital signs were stable throughout and after the procedure. The Glasgow Coma Score shortly after TGA onset was E4M6V5. In addition, the patient showed neither focal neurologic symptoms nor convulsions or headache. 

Serum electrolytes, glucose, and hemogram were within the normal range. Brain computed tomography was performed immediately and revealed no specific abnormality. Brain magnetic resonance imaging (MRI) performed 8 hours after symptom onset showed no abnormalities as well as the MR angiography; also, the diffusion-weighted image (DWI) did not reveal abnormal enhancement.

There was no significant atherosclerosis in the transcranial duplex sonography and no abnormal epileptiform discharge at electroencephalogram. Echocardiography performed both immediately after procedure and thereafter revealed preserved left ventricular function and normal cardiac structures.

The patient became re-orientated and recalled his lost memory about 8-9 hours after symptom onset. He had no neurologic deficits either at the time of discharge or during outpatient department follow-up for 8 months.

## Discussion

Several differential diagnoses for TGA should be considered [2,3]. First, our patient remained attentive and could be communicated with, in contrast to the confused or distracted status of acute delirium, or the automatism or blank stare of the postictal state after complex partial seizure. Second, our patient recovered from amnesia after 8-9 hours, unlike the temporal lobe epilepsy, which also causes amnesia but often lasts less than one hour per episode. In addition, temporal lobe epilepsy tends to be recurrent but TGA does not. Third, unlike transient ischemic attack (TIA), TGA involves only transient antegrade amnesia and some recent retrograde amnesia, while TIA is often accompanied by motor or sensory symptoms. TGA has a low recurrence rate and tends to have no stroke risk factors, whereas TIA tends to be recurrent, accompanied by stroke risk factors and tends to increase the risk of subsequent stroke. Fourth, psychogenic amnesia (PA) might be considered. It usually precipitated by a specific psychosocial stressor. In addition, the personal recognition in PA is impaired and the memory loss is mainly retrograde.

Many mechanisms have been suggested for TGA, such as a thromboembolic event, compromised hemodynamics, migraine attack, or the cerebral congestion associated with jugular venous valve dysfunction. Embolic events often result in stroke or TIA but not TGA except in very rare cases in which the emboli plug the posterior cerebral, anterior choroidal or thalamic penetrating arteries and causing only the amnesia (i.e., amnesic stroke) [4]. However, there are typical stroke findings at MRI and the amnesia will take days for resolution. Because the catheter was manipulated in the left heart, even the preexisting or fresh thrombus or tissue debris might be spread distally. In order to minimize the risk of emboli spread, to ensure proper anticoagulation is important. Although some authors reported subclinical intracranial embolic events detected by the MRI before and after pulmonary vein ablation even under adequate anticoagulation, they were free of symptoms [5].

The vital signs of our patient were stable throughout the procedure. There was no stenosis over the carotid arteries and no deterioration of left ventricular function. The possibility of hemodynamic disturbance to influence cerebral perfusion is less likely. Although migraine could be associated with TGA; our patient had no history and no headache at any time during hospitalization. Valsalva-like maneuvers could be attributed to TGA in susceptible individuals with internal jugular vein valve dysfunction such as during sexual intercourse, strenuous exercise, or sudden immersion into cold water (i.e., diving reflex) [6]. There are still debates on this theory. Currently, there is no confirmed mechanism attributable to TGA. Rather than a single, vascular-specific etiology, TGA should be considered a transient disturbance in the hippocampus (especially CA1 neurons) caused by a multifactorial, non-vascular etiology.

TGA is mainly a clinical diagnosis, brain MRI performed 24-72 hours after symptom onset could be helpful. Characteristic MRI findings include reversible small punctate DWI lesions over the temporal lobe, thalamus, or cerebellum and especially the hippocampus. In those patients who have positive DWI findings, the findings could be captured mostly within 12-72 hours after symptom onset with a higher prevalence of 57% [7,8]. Although the recurrence of TGA is less frequent, patients with recurrent TGA have a higher prevalence of reversible DWI lesions [9].

## Conclusion

TGA is a rare complication of electrophysiologic procedures. The exact etiology of TGA is still unknown. Some patients exhibit reversible high-density hippocampal lesions on DWI, although some patients do not. TGA does not require specific treatment but several serious differential diagnoses should be excluded. The general long-term outcome of patients with TGA appears to be benign.

## Figures and Tables

**Figure 1A:**
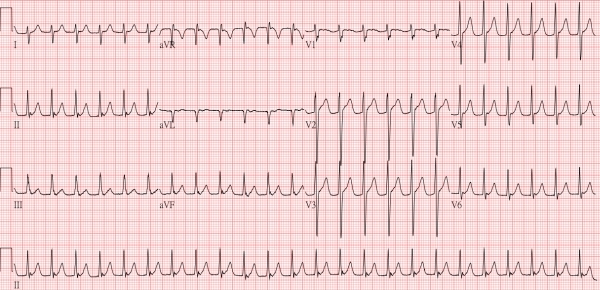
12-lead electrocardiogram showing paroxysmal supraventricular tachycardia

**Figure 1B:**
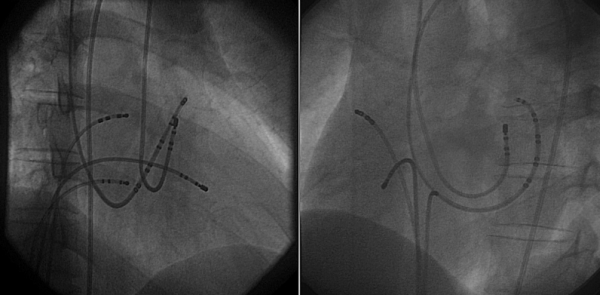
Successful sites of ablation (view of RAO 30º and LAO 60º)
